# Antimicrobial Ointments and Methicillin-Resistant *Staphylococcus aureus* USA300

**DOI:** 10.3201/eid1710.101365

**Published:** 2011-10

**Authors:** Masahiro Suzuki, Kazuhiro Yamada, Miki Nagao, Etsuko Aoki, Masakado Matsumoto, Tatsuya Hirayama, Hiroaki Yamamoto, Reiji Hiramatsu, Satoshi Ichiyama, Yoshitsugu Iinuma

**Affiliations:** Author affiliations: Aichi Prefectural Institute of Public Health, Nagoya, Japan (M. Suzuki, K. Yamada, M. Matsumoto, T. Hirayama, H. Yamamoto, R. Hiramatsu);; Kyoto University Hospital, Kyoto, Japan (M. Nagao, S. Ichiyama, Y. Iinuma);; Kyoto University, Kyoto (M. Nagao, S. Ichiyama, Y. Iinuma);; National Hospital Organization Nagoya Medical Center, Nagoya (E. Aoki);; Kanazawa Medical University, Kahoku, Japan (Y. Iinuma)

**Keywords:** bacteria, antimicrobial drug resistant, methicillin-resistant Staphylococcus aureus, MRSA, over-the-counter, community acquired infections, nonprescription drugs, ointments, bacitracin, neomycin, polymyxin B, drug resistance, USA300, dispatch

## Abstract

We tested 259 methicillin-resistant *Staphylococcus aureus* isolates and 2 USA300 ATCC type strains for susceptibility to bacitracin and neomycin contained in over-the-counter antibacterial ointments. Resistance to both bacitracin and neomycin was found only in USA300. The use of over-the counter antimicrobial drugs may select for the USA300 clone.

Community-acquired methicillin-resistant *Staphylococcus aureus* (CA-MRSA) is rapidly spreading worldwide. MRSA USA300 is a clone of increasing public health concern among rapidly disseminating CA-MRSA strains in the United States (*1*). MRSA USA300 is designated as sequence type (ST) 8 by multilocus sequence typing (MLST) and possesses staphylococcal cassette chromosome *mec* (SCC*mec*) type IVa. Although the rapid dissemination of the USA300 clone may occur because of a high virulence level that arises from the production of Panton-Valentine leukocidin (PVL) or an existing arginine catabolic mobile element (ACME) (*2*), there is no conclusive evidence to support this hypothesis (*3*). Furthermore, the hypothesis cannot account for the rapid dissemination of MRSA in countries where USA300 clones are not the dominant clones (most European countries, South Korea, and Japan) (*4**–**6*). In most European countries, the dominant CA-MRSA clone is the European clone (ST80, SCC*mec* type IV, PVL positive and ACME negative) (*1**,**4*). In South Korea, only 1 isolate was a USA300 clone among 138 MRSA isolates collected from patients with bacteremia and soft tissue infection (*5*). In Japan, the MRSA USA300 clone is rare (*6*).

In many cases, soft tissue infection acquired in communities was treated by using over–the-counter (OTC) drugs called triple-antibiotic ointment (TAO), e.g., Neosporin (polymyxin B [PL-B] sulfate, 5,000 units/g; bacitracin, 400 units/g; and neomycin, 3.5 mg/g) and Polysporin triple ointment (PL-B sulfate, 10,000 units/g; bacitracin, 500 units/g; and gramicidin 0.25 mg/g). These ointments contain antimicrobial drugs at concentrations far exceeding their MICs among *S. aureus* strains (16–32 μg/mL [equivalent to 124–248 unit/mL] for PL-B, <1–64 units/mL for bacitracin, and <1–>128 μg/mL for neomycin) (*7**,**8*). It is hypothesized that CA-MRSA cases in the United States were under the selective pressure of TAOs.

In this study, we tested the susceptibilities of MRSA isolates, including the USA300 clone, to the antimicrobial drugs in TAOs. We also considered the possible role of TAOs in spreading the USA300 clone.

## The Study

We selected 222 MRSA isolates that were not classified as the New York/Japan (NY/JP) clone on the basis of the absence of SCC*mec kdpC* (*9*). In addition, 37 NY/JP clone-like isolates were used. A total of 259 MRSA isolates were tested in our study. Of these 259 isolates, 227 were collected during 2004–2010 at Nagoya Medical Center, and 32 isolates were collected in 2006–2009 at Kyoto University Hospital, including 9 USA300 outbreak isolates (*6*). Details of isolates used in this study are shown in Table 1. ATCC BAA1556 (USA300 FPR3757) (American Type Culture Collection, Manassas, VA, USA) and ATCC BAA1717 (USA300-HOU-MR TCH1516) strains were also used in our study. Susceptibilities to bacitracin and neomycin were tested by the Kirby-Bauer disk diffusion method (Becton Dickinson, Franklin Lakes, NJ, USA). MICs of bacitracin, neomycin, and PL-B for USA300 strains were determined by the agar dilution method according to the Clinical and Laboratory Standard Institute M07-A8 guidelines (*10*). To observe interaction among these 3 antimicrobial drugs, a double-disk synergy test was performed with modification by using ATCC BAA1717 (*11*).

**Table 1:** Source of methicillin-resistant *Staphylococcus aureus* isolates, Japan, 2004–2010

Source	Outpatients			Inpatients		Health care workers
	No. community-acquired infections*	No. hospital-acquired infections		No. community-acquired infections*	No. hospital-acquired infections	
Skin and soft tissue	23	7		4	23	0
Bloodstream	0	2		1	19	0
Respiratory tract	0	2		1	17	0
Urinary tract	0	2		2	3	0
Ear	8	0		0	1	0
Eye	3	0		0	3	0
Others	1	1		2	3	0
Carriage	14	10		3	29	0
Screening	50	0		2	20	3
Total	99	24		15	118	3

*Community-acquired infections were determined on the basis of patients’ histories according to Centers for Disease Control and Prevention (Atlanta, GA, USA) guidelines (www.cdc.gov/mrsa/diagnosis/index.html).

SCC*mec* were determined according to the method of Hisata et al. (*12*). Isolates possessing both PVL and *arcA* (*13*) were analyzed by pulsed-field gel electrophoresis as described in our previous study (*9*). Moreover, USA300 isolates were genotyped by MLST (www.mlst.net) and staphylococcal protein A (*spa*) typing (www.spaserver.ridom.de).

Nineteen of the 259 isolates harbored both the PVL and the *arcA* gene. Of these 19 isolates, 18 had been collected from Kyoto University Hospital and 1 from Nagoya Medical Center (Table 2). All 19 PVL- and ACME-positive isolates were determined to be ST8 by MLST. These isolates showed USA300 PFGE patterns identical to ATCC BAA1556 and were of SCC*mec* type IVa. SCC*mec* elements of other isolates were determined as type I (n = 4), IIa (n = 37), IIb (n = 52), II untypeable (n = 14), IV (n = 104), and V (n = 9). The SCC*mec* element of the remaining 20 isolates could not be identified.

**Table 2:** Bacitracin and neomycin susceptibility of MRSA USA300 and other MRSA isolates*

Bacitracin/neomycin	MRSA USA300 (MICs of bacitracin, neomycin, and polymyxin B)†			Other MRSA
Kyoto University Hospital	Nagoya Medical Center	ATCC type strains
R/R	9 (400, 128, 200–400)	0	BAA1717 (400, 128, 400)	0
R/S	0	1 (400, 0.25, 400)		0
S/R	0	0		11
I/S	0	0		1
S/I	0	0		132
S/S	9 (6.25–12.5, 0.25, 400)	0	BAA1556 (6.25, 0.25, 400)	96
Total	18	1	2	240

*MRSA, methicillin resistant *Staphylococcus aureus*; ATCC, American Type Culture Collection (Manassas, VA, USA); R, resistant; S, susceptible; I, intermediate resistance. †MICs are expressed as units/mL for bacitracin and polymyxin B and in μg/mL for neomycin.

The 18 USA300 isolates collected from Kyoto University Hospital showed the same *spa* type (t008). However, the 1 USA300 isolate collected from Nagoya Medical Center was of *spa* t190.

ATCC BAA1717 and 9 USA300 isolates collected during 2007–2009 at Kyoto University Hospital were resistant to both bacitracin and neomycin. The USA300 isolate detected at Nagoya Medical Center in 2004 was bacitracin resistant and neomycin susceptible. The other 9 USA300 isolates and ATCC BAA1556 were susceptible to both drugs (Table 2). Highlander et al. (*14*) found that the bacitracin- and aminoglycoside-resistant genes were located on pUSA300-HOU-MR, a plasmid typically observed in the USA300 strain TCH1516. The resistance to bacitracin and neomycin may depend on the presence of the plasmid and may be absent in some USA300 clones.

On the other hand, nearly all MRSA isolates that were determined to be a type other than USA300 were susceptible to bacitracin. One isolate was determined to have intermediate resistance to bacitracin. Also, 11 (4.5%) of the 240 MRSA isolates not deemed to be USA300 were resistant to neomycin, while 132 (55%) demonstrated intermediate resistance (Table 2). A study performed in the 1990s reported that most MRSA strains were susceptible to bacitracin, and many were resistant to neomycin (*8*). Our findings were consistent with the previous study.

MICs of bacitracin, neomycin, and PL-B were 400 units/mL, 128 μg/mL, and 400 units/mL, respectively, among most USA300 isolates with resistance to both bacitracin and neomycin (Table 2). The concentrations of neomycin and PL-B in the TAOs were ≈10 to 30× higher than the MICs of both drugs. In addition, neomycin and PL-B were observed to be weakly synergistic (Figure). However, Bearden et al. reported that despite containing antimicrobial drugs at concentrations far exceeding their MICs among MRSA, PL-B and neomycin ointment, or PL-B and gramicidin ointment exhibited deficient bactericidal activity in time-kill assays (*15*). Bacitracin may thus be required for sufficient bactericidal activity. Acquiring resistance to bacitracin and neomycin may be essential for survival under the selective pressure of TAOs. If so, bacitracin resistance should be considered a key characteristic of the USA300 clone.

**Figure:**
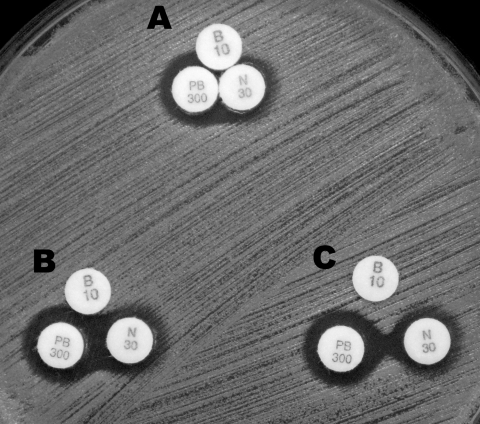
Double-disk synergy test with 3 disks, bacitracin (B10 disk), neomycin (N30 disk), or polymyxin B (PL-B, PB300 disk) was performed with USA300 strain ATCC BAA1717. Disks were placed at 6 mm (A), 9 mm (B), and 11 mm (C) distance from disk centers. Neomycin and PL-B were found to be weakly synergistic.

TAOs containing bacitracin, neomycin, and PL-B are widely used in the United States; thus, bacitracin- and neomycin-resistant strains may be selected by the selective pressure of the TAOs. Although bacitracin and neomycin ointments are also available as OTC drugs in Japan, use of the ointments is not widespread. As a result, the selective pressure that leads to bacitracin and neomycin resistance is weak in Japan.

## Conclusions

The emergence of MRSA USA300 depends partly on the virulence of MRSA USA300, but it may be influenced by usage of OTC drugs. In each country, susceptibilities of MRSA USA300 to bacitracin and neomycin should be thoroughly investigated, and relationships between the dissemination of MRSA USA300 and the usage of OTC drugs should be clarified. Such an investigation will provide valuable information regarding the emergence of organisms resistant to OTC topical antibiotics and likely a warning against the indiscriminate use of antimicrobial drugs. Further studies are required to validate these findings.
